# Vagus Nerve Stimulation Paired With Rehabilitation for Upper Limb Motor Impairment and Function After Chronic Ischemic Stroke: Subgroup Analysis of the Randomized, Blinded, Pivotal, VNS-REHAB Device Trial

**DOI:** 10.1177/15459683221129274

**Published:** 2022-10-13

**Authors:** Jesse Dawson, Navzer D. Engineer, Steven C. Cramer, Steven L. Wolf, Rushna Ali, Michael W. O’Dell, David Pierce, Cecília N. Prudente, Jessica Redgrave, Wuwei Feng, Charles Y. Liu, Gerard E. Francisco, Benjamin L. Brown, Anand Dixit, Jen Alexander, Louis DeMark, Vibor Krishna, Steven A. Kautz, Arshad Majid, Brent Tarver, Duncan L. Turner, Teresa J. Kimberley

**Affiliations:** 1 School of Cardiovascular and Metabolic Health, College of Medical, Veterinary and Life Sciences, University of Glasgow, Glasgow, UK; 2 MicroTransponder Inc., Austin, TX, USA; 3 Department of Neurology, David Geffen School of Medicine at UCLA, and California Rehabilitation Institute; Los Angeles, CA, USA; 4 Division of Physical Therapy, Department of Rehabilitation Medicine, Emory University School of Medicine, Atlanta, GA, USA; 5 Department of Neurosciences, Spectrum Health, Grands Rapids, MI, USA; 6 Clinical Rehabilitation Medicine, Weill Cornell Medicine, New York City, NY, USA; 7 Sheffield Institute for Neurological Sciences (SITraN), Sheffield, UK; 8 Department of Neurology, Duke University School of Medicine, Durham, NC, USA; 9 USC Neurorestoration Center and Department of Neurological Surgery, USC Keck School of Medicine, Los Angeles, CA, USA, and Rancho Los Amigos National Rehabilitation Center, Downey, CA, USA; 10 Department of Physical Medicine and Rehabilitation, The University of Texas Health Science Center McGovern Medical School, and The Institute for Rehabilitation and Research (TIRR) Memorial Hermann Hospital; Houston, TX, USA; 11 Department of Neurosurgery, Ochsner Neuroscience Institute, Covington, Los Angeles, USA; 12 Stroke Service, The Newcastle Upon Tyne Hospitals NHS Foundation Trust, Newcastle upon Tyne, UK; 13 Brooks Rehabilitation, Jacksonville, FL, USA; 14 Department of Neurosurgery, University of North Carolina, Chapel Hill, NC, USA; 15 Ralph H. Johnson VA Medical Center, Charleston, SC, USA and Department of Health Sciences and Research, Medical University of South Carolina, Charleston, SC, USA; 16 Sheffield Institute for Neurological Sciences (SITraN) and Sheffield Teaching Hospitals, Sheffield, UK; 17 School of Health, Sport and Bioscience, University of East London, London, UK; 18 Department of Physical Therapy, MGH Institute of Health Professions, Boston, MA, USA

**Keywords:** neuromodulation, rehabilitation, stroke, upper extremity

## Abstract

**Background:**

Vagus Nerve Stimulation (VNS) paired with rehabilitation improved upper extremity impairment and function in a recent pivotal, randomized, triple-blind, sham-controlled trial in people with chronic arm weakness after stroke.

**Objective:**

We aimed to determine whether treatment effects varied across candidate subgroups, such as younger age or less injury.

**Methods:**

Participants were randomized to receive rehabilitation paired with active VNS or rehabilitation paired with sham stimulation (Control). The primary outcome was the change in impairment measured by the Fugl–Meyer Assessment Upper Extremity (FMA-UE) score on the first day after completion of 6-weeks in-clinic therapy. We explored the effect of VNS treatment by sex, age (≥62 years), time from stroke (>2 years), severity (baseline FMA-UE score >34), paretic side of body, country of enrollment (USA vs UK) and presence of cortical involvement of the index infarction. We assessed whether there was any interaction with treatment.

**Findings:**

The primary outcome increased by 5.0 points (SD 4.4) in the VNS group and by 2.4 points (SD 3.8) in the Control group (*P* = .001, between group difference 2.6, 95% CI 1.03-4.2). The between group difference was similar across all subgroups and there were no significant treatment interactions. There was no important difference in rates of adverse events across subgroups.

**Conclusion:**

The response was similar across subgroups examined. The findings suggest that the effects of paired VNS observed in the VNS-REHAB trial are likely to be consistent in wide range of stroke survivors with moderate to severe upper extremity impairment.

## Introduction

Stroke is a leading cause of adult disability. Upper limb impairment and inability to effectively use the arm and hand for functional daily tasks are common and persists in approximately half of people who have upper limb impairment at onset.^1^ These limitations have a detrimental impact on quality of life and improving upper limb impairment and function are a priority for stroke survivors.^2^

The use of vagus nerve stimulation (VNS) paired with rehabilitation to improve moderate to severe upper limb motor deficits associated with chronic ischemic stroke was recently approved the U.S. Food and Drug Administration. VNS augments task specific neuroplasticity by providing rapid cholinergic, noradrenergic, and serotonergic modulation.^3^ VNS paired with rehabilitation leads to greater recovery of forelimb function in rodent models than either motor training or VNS alone.^3,4^ A combined analysis of data from 2 pilot feasibility trials of VNS paired with rehabilitation therapy^5,6^ found an improvement in impairment following VNS in people with long-term arm weakness after ischemic stroke.^7^ In the recently published pivotal VNS-REHAB trial, there was a significant difference in change in Fugl–Meyer Assessment Upper Extremity (FMA-UE) score in favor of paired VNS following 6 weeks of in-clinic therapy.^8^ There was also a higher clinically important response rate, defined as a greater than 6-point improvement in the FMA-UE score, and improvements in functional measures with paired VNS at 90 days after completion of in-clinic therapy. However, participants did not have a uniform response to VNS, so identifying those with a higher chance of responding could optimize prescription of this therapy. Pooled analysis of data from both pilot trials did not find any clear relationship between baseline variables and change in FMA-UE score with VNS, although lower baseline Fugl–Meyer score was associated with greater improvement across both treatment groups.^7^ However, this analysis was based on a small sample size.

Here we perform a post-hoc subgroup analysis of data from the VNS-REHAB trial. We identified variables of interest based on known predictors of upper limb outcome. We examined whether the effect of paired VNS treatment differs by reported sex, age, time from stroke, severity of upper limb impairment, country of enrollment, paretic side, and whether there was cortical involvement of the index infarction.

## Methods

The design and methods of the VNS-REHAB trial have been previously described.^9^ The trial was a randomized, blinded, sham-controlled clinical trial conducted at 19 stroke rehabilitation centers in the USA and UK. A total of 108 participants were enrolled between Oct 2, 2017, and Sept 12, 2019. Participants with moderate-to-severe arm weakness, at least 9 months after ischemic stroke, were randomly assigned (1:1) to either rehabilitation paired with active VNS (0.8 mA, 100 μs, 30 Hz stimulation pulses, lasting 0.5 seconds) or rehabilitation paired with sham stimulation (0.0 mA). Participants received 6 weeks of in-clinic therapy (3 times per week; total of 18 sessions) followed by a home exercise program. All participants gave full informed consent. The trial was approved by the regulatory authorities and ethics committees/institutional review boards in the US and UK.

### Outcome Measures

The primary outcome for this analysis was the change in impairment measured by the FMA-UE score after completion of the 6-weeks in-clinic therapy. Secondary endpoints were change in FMA-UE score and Wolf Motor Function Test (WMFT) at day-90 after completion of in-clinic therapy, and clinically important response rate, defined as a greater than or equal to 6-point change in FMA-UE score at 90 days after in-clinic therapy^10^ and a greater than 0.4-point change on the WMFT.^11^ We also assessed occurrence of serious adverse events and adverse events related to device implantation and device use.

### Subgroups of Interest

We identified the following variables of interest: reported age, sex (male vs female), country of enrollment (UK vs USA), severity of upper limb impairment, time since stroke, paretic side (right vs left) and whether there was cortical involvement of the index infarct. Severe upper limb impairment, time from stroke and age were dichotomized based on whether the baseline value was above or below the median (FMA-UE score of 34, time from stroke 2 years and age 62 years). Presence of cortical involvement was visually confirmed by a trained image analyst using data from the study specific MRI performed before VNS device implantation.

### Statistical Analysis

All analyses used the intention-to-treat population. For the analysis of paretic side, only right-handed participants were included as the majority of participants were right hand dominant (48/53 [90.6%] in the VNS group and 50/55 [91%] in the control group). We calculated the mean difference and 95% CI for the FMA-UE and WMFT and the difference in response rates between active VNS and sham stimulation in the entire population and then by subgroup. We then assessed whether there was any interaction between subgroup factor and the mean difference in FMA-UE score at day 90 using a two-way ANOVA model including treatment and subgroup as factors. A *P* value of <.05 was used to determine statistical significance.

## Results

About 108 participants were randomized to either the active VNS group (n = 53) or the sham stimulation group (n = 55). Baseline characteristics are shown in Table 1.

**Table 1.** Baseline Demographic and Clinical Characteristics by Randomization Group in the Intention-to-Treat Population.

	VNS (n = 53)	Control (n = 55)
Sex (N, %)		
Male	34 (64%)	36 (65.5%)
Female	19 (37%)	19 (35%)
Ethnicity (N, %)		
Caucasian	42 (79%)	43 (78%)
African American	9 (17%)	9 (16%)
Asian, Indian, Other	1 (2%)	4 (7%)
Not reported	1 (2%)	1 (2%)
Age (y, mean ± SD)	59.1 ± 10.2	61.1 ± 9.2
Time since stroke (y)	3.1 ± 2.3	3.3 ± 2.6
Handedness (right/left/ambidextrous)	48 (91%)/4 (8%)/1 (2%)	50 (91%)/5 (9)/0
Side of paresis (right)	25 (47%)	26 (47%)
FMA-UE baseline score (mean ± SD)	34.4 ± 8.2	35.7 ± 7.8
WMFT functional score	2.71 ± 0.70	2.83 ± 0.65

Abbreviations: VNS, vagus nerve stimulation; SD, standard deviation; FMA-UE, Fugl–Meyer Assessment Upper Extremity; WMFT, Wolf Motor Function Test.

Participants Could Select More Than One Option for Ethnicity. FMA-UE is Out of 66 Maximum Points, With Higher Scores Meaning Better Motor Status.

**Figure 1.**
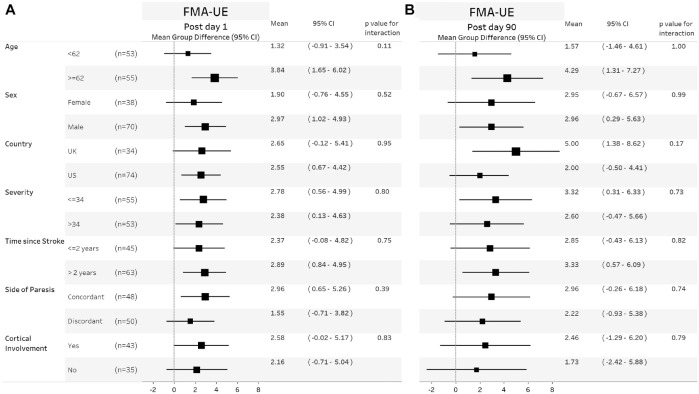
Forest plots showing mean group difference for FMA-UE change in score at day 1 (A) and day 90 (B) post completion of in-clinic therapy across different subgroups. Within each subgroup category, black square shows the mean group difference, and the size of the squares represents the degree of change. Horizontal lines denote 95% confidence. *P* values are given for the test of interaction between the group difference and subgroup of interest. Values to the right of the zero vertical line show a between group difference in favor of VNS. Abbreviation: FMA-UE, Fugl–Meyer Assessment Upper Extremity; VNS, vagus nerve stimulation.

**Figure 2.**
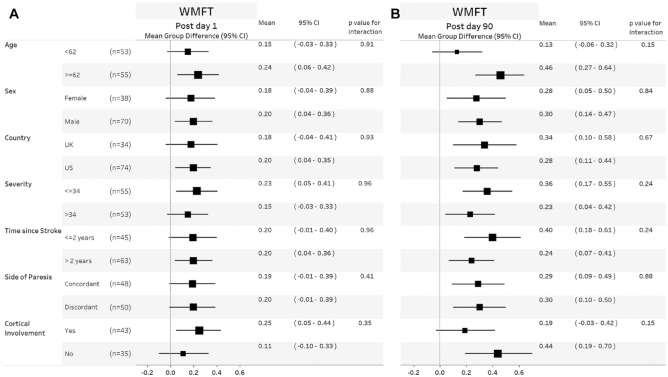
Forest plots showing mean group difference for WMFT change in score at day 1 (A) and day 90 (B) post completion of in-clinic therapy across different subgroups. Within each subgroup category, black square shows the mean group difference, and the size of the squares represents the degree of change. Horizontal lines denote 95% confidence. *P* values are given for the test of interaction between the group difference and subgroup of interest. Values to the right of the zero vertical line show a between group difference in favor of VNS. Values to the right of the vertical line show a between group difference in favor of VNS. Abbreviations: WMFT, Wolf Motor Function Test; VNS, vagus nerve stimulation.

**Figure 3.**
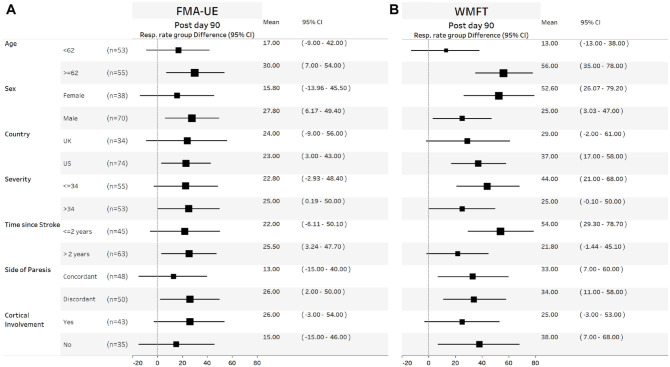
Forest plots showing mean group response rate difference for FMA-UE (A) and WMFT (B) at day 90 post completion of in-clinic therapy across different subgroups. Within each subgroup category, black square shows the mean group difference, and the size of the squares represents the size of the response difference. Horizontal lines denote 95% confidence. Values to the right of the zero vertical line show a between group absolute response rate difference in favor of VNS. Abbreviations: FMA-UE, Fugl–Meyer Assessment Upper Extremity; WMFT, Wolf Motor Function Test; VNS, vagus nerve stimulation; resp, response.

On the first day after completion of in-clinic therapy, the mean (±standard deviation) FMA-UE score increased by 5.0 points (4.4) in the VNS group and by 2.4 points (3.8) in the Control group (*P* = .001, between group difference 2.6, 95% CI 1.03-4.2). This difference was similar across subgroups of age, sex, country, stroke severity, time from stroke, paretic side, and presence of cortical involvement (Figure 1A). The between group difference in FMA-UE score at day-90 was 3.0 points (95% CI 0.8-5.1) and was similar across subgroups (Figure 1B). The between group difference in WMFT at day-90 was 0.3 points (95% CI 0.2-0.4) and was similar across subgroups (Figure 2B). The between group difference in response rates on the FMA-UE score and WMFT were 24% (95% CI 6%-41%) and 35% (95% CI 18%-52%), respectively, and were similar across subgroups (Figure 3). No significant treatment interactions were observed with change in FMA-UE or WMFT score and subgroups of interest at either day 1 or day-90 post completion of in-clinic therapy (all *P* > .05).

There were 15 reported SAEs in 12 participants. None were rated as related to study treatment or device implantation according to the investigators. There were 335 adverse events reported. The frequency of these events by subgroup is given in Table 2.

**Table 2.** Table Shows the Number of Events Within Each Subgroup.

	No. of participants (n)	SAE, n = 15 events	AE related to device implantation, n = 51 events
Age, y			
<62	53	2	30
≥62	55	13	21
Sex			
Male	70	6	35
Female	38	9	16
Country of enrollment			
USA	74	13	30
UK	34	2	21
Baseline FMA-UE score			
≤34	55	9	24
>34	53	6	27
Time since stroke, y			
≤2	45	7	19
>2	63	8	32
Paretic side (right dominant)			
Right	48	6	28
Left	50	9	23
Cortical involvement			
Yes	43	5	20
No	35	5*	17*

Abbreviations: SAE, serious adverse event; AE, adverse event; FMA-UE, Fugl–Meyer Assessment Upper Extremity.

* There were a further 5 SAEs and 15 further AEs in people who did not undergo the study specific MRI scan.

## Discussion

We observed that the beneficial effects of paired VNS on measures of upper extremity impairment and function in people with chronic ischemic stroke were consistent across subgroups including measures of age, sex, impairment severity, time from stroke, stroke location, and paretic side. Our findings suggest the response to VNS therapy is consistent across the range of participants who met the trial eligibility criteria.

Paired VNS uses an implantable VNS device and is performed under general anesthesia. As with any device implant, there are associated risks. There is a reported rate of transient vocal cord palsy of approximately 3% following all types of VNS device implantations.^12^ We saw no evidence of a difference in rate of reported (unrelated) serious adverse events and adverse events due to implantation by subgroup.

Our findings are consistent with a previous analysis of data from pilot studies of paired VNS therapy^7^ and from studies exploring predictors of constraint-induced movement therapy where there is little evidence of a difference in response across subgroups.^13^ In addition, pre-clinical studies of VNS paired with rehabilitation have also shown a consistent effect in different experimental models of aged versus young animals, cortical and subcortical infarction, and intracerebral hemorrhage.^4,14,15^ Although there are well described predictors of upper limb recovery after stroke, such as age, sex, lesion site, severity of motor impairment, and measures of evoked potentials identified in a large systematic review and meta-analysis,^16^ studies of response to specific interventions were excluded. Recent studies have explored the predictive ability of biomarker-based algorithms to predict recovery of upper limb function when used within days of stroke.^17^ These data incorporate measures of stroke severity and movement and motor evoked potentials but whether such tools can predict response to paired VNS therapy in chronic stroke requires further exploration. Time from onset of stroke and degree of impairment have been associated with response to robotic therapy in subacute stroke,^18^ but not with more chronic impairment. Corticospinal tract excitability has been associated with response to robotic training after chronic stroke^19^ and the degree of injury to the corticospinal tract is associated with response to therapy.^20^ We did not gather data on corticospinal tract excitability in our study, so we cannot assess whether this factor would be associated with response to VNS.

This analysis has additional limitations. The small sample size constrains our ability to identify small but potentially important differences between subgroups and precludes us from further dividing the groups (ie, older females vs older males). In addition, the variables we have assessed are not exhaustive and there remain several variables of interest. We defined stroke severity according to the median of the baseline FMA-UE score within our study population. Other studies suggest that a score of greater than 31 corresponds with poor arm capacity on the Action Research Arm test score.^21^ We therefore feel the median values is clinically informative for analyses, but we have not explored other cut-offs. We excluded people with significant sensory impairment from the study so cannot assess whether such people would benefit. In addition, people with a FMA-UE score of <20 were not included. Therefore, although we saw no evidence of a differential effect by stroke severity, we have not as yet acquired data on the most severely affected participants. This was also a post hoc analysis.

In summary, the response to VNS treatment was similar across subgroups of interest and there was no obvious important difference in rates of adverse events across subgroups. These findings suggest that the effect of VNS observed in the VNS-REHAB trial^8^ are likely to be consistent in a wide range of stroke survivors with moderate to moderately severe upper extremity impairment. This should be clarified by further studies.

## References

[1] Kelly-Hayes M Beiser A Kase CS Scaramucci A D’Agostino RB Wolf PA. The influence of gender and age on disability following ischemic stroke: the Framingham study. J Stroke Cerebrovasc Dis. 2003;12(3):119-126. 17903915 10.1016/S1052-3057(03)00042-9

[2] Pollock A St George B Fenton M Firkins L. Top 10 research priorities relating to life after stroke–consensus from stroke survivors, caregivers, and health professionals. Int J Stroke. 2014;9(3):313-320. 23227818 10.1111/j.1747-4949.2012.00942.x

[3] Porter BA Khodaparast N Fayyaz T , et al. Repeatedly pairing vagus nerve stimulation with a movement reorganizes primary motor cortex. Cereb Cortex. 2012;22(10):2365-2374. 22079923 10.1093/cercor/bhr316

[4] Khodaparast N Kilgard MP Casavant R , et al. Vagus nerve stimulation during rehabilitative training improves forelimb recovery after chronic ischemic stroke in rats. Neurorehabil Neural Repair. 2016;30(7):676-684. 26542082 10.1177/1545968315616494 PMC4854844

[5] Dawson J Pierce D Dixit A , et al. Safety, feasibility, and efficacy of vagus nerve stimulation paired with upper-limb rehabilitation after ischemic stroke. Stroke. 2016;47(1):143-150. 26645257 10.1161/STROKEAHA.115.010477 PMC4689175

[6] Kimberley TJ Pierce D Prudente CN , et al. Vagus nerve stimulation paired with upper limb rehabilitation after chronic stroke. Stroke. 2018;49(11):2789-2792. 30355189 10.1161/STROKEAHA.118.022279

[7] Dickie DA Kimberley TJ Pierce D Engineer N Tarver WB Dawson J. An exploratory study of predictors of response to vagus nerve stimulation paired with upper-limb rehabilitation after ischemic stroke. Sci Rep. 2019;9(1):15902. 31685853 10.1038/s41598-019-52092-x PMC6828969

[8] Dawson J Liu CY Francisco GE , et al. Vagus nerve stimulation paired with rehabilitation for upper limb motor function after ischaemic stroke (VNS-REHAB): a randomised, blinded, pivotal, device trial. Lancet. 2021;397(10284):1545-1553. 33894832 10.1016/S0140-6736(21)00475-X PMC8862193

[9] Kimberley TJ Prudente CN Engineer ND , et al. Study protocol for a pivotal randomised study assessing vagus nerve stimulation during rehabilitation for improved upper limb motor function after stroke. Eur Stroke J. 2019;4(4):363-377. 31903435 10.1177/2396987319855306 PMC6921938

[10] Page SJ Fulk GD Boyne P. Clinically important differences for the upper-extremity Fugl-Meyer Scale in people with minimal to moderate impairment due to chronic stroke. Phys Ther. 2012;92(6):791-798. 22282773 10.2522/ptj.20110009

[11] Lin KC Hsieh YW Wu CY Chen CL Jang Y Liu JS. Minimal detectable change and clinically important difference of the Wolf Motor Function Test in stroke patients. Neurorehabil Neural Repair. 2009;23(5):429-434. 19289487 10.1177/1545968308331144

[12] Kahlow H Olivecrona M. Complications of vagal nerve stimulation for drug-resistant epilepsy: a single center longitudinal study of 143 patients. Seizure. 2013;22(10):827-833. 23867218 10.1016/j.seizure.2013.06.011

[13] Kwakkel G Veerbeek JM van Wegen EE Wolf SL. Constraint-induced movement therapy after stroke. Lancet Neurol. 2015;14(2):224-234. 25772900 10.1016/S1474-4422(14)70160-7 PMC4361809

[14] Hays SA Ruiz A Bethea T , et al. Vagus nerve stimulation during rehabilitative training enhances recovery of forelimb function after ischemic stroke in aged rats. Neurobiol Aging. 2016;43:111-118. 27255820 10.1016/j.neurobiolaging.2016.03.030 PMC5206764

[15] Hays SA Khodaparast N Hulsey DR , et al. Vagus nerve stimulation during rehabilitative training improves functional recovery after intracerebral hemorrhage. Stroke. 2014;45(10):3097-3100. 25147331 10.1161/STROKEAHA.114.006654 PMC4175144

[16] Coupar F Pollock A Rowe P Weir C Langhorne P. Predictors of upper limb recovery after stroke: a systematic review and meta-analysis. Clin Rehabil. 2012;26(4):291-313. 22023891 10.1177/0269215511420305

[17] Stinear CM Byblow WD Ackerley SJ Smith MC Borges VM Barber PA. PREP2: a biomarker-based algorithm for predicting upper limb function after stroke. Ann Clin Transl Neurol. 2017;4(11):811-820. 29159193 10.1002/acn3.488 PMC5682112

[18] Lee JJ Shin JH. Predicting clinically significant improvement after robot-assisted upper limb rehabilitation in subacute and chronic stroke. Front Neurol. 2021;12:668923. 34276535 10.3389/fneur.2021.668923 PMC8281036

[19] Milot MH Spencer SJ Chan V , et al. Corticospinal excitability as a predictor of functional gains at the affected upper limb following robotic training in chronic stroke survivors. Neurorehabil Neural Repair. 2014;28(9):819-827. 24642382 10.1177/1545968314527351 PMC4167999

[20] Cassidy JM Tran G Quinlan EB Cramer SC. Neuroimaging identifies patients most likely to respond to a restorative stroke therapy. Stroke. 2018;49(2):433-438. 29321336 10.1161/STROKEAHA.117.018844 PMC5780222

[21] Hoonhorst MH Nijland RH van den Berg JS Emmelot CH Kollen BJ Kwakkel G. How do Fugl-Meyer arm motor scores relate to dexterity according to the action research arm test at 6 months poststroke? Arch Phys Med Rehabil. 2015;96(10):1845-1849. 26143054 10.1016/j.apmr.2015.06.009

